# Tagging and catching: rapid isolation and efficient labeling of organelles using the covalent Spy-System in planta

**DOI:** 10.1186/s13007-020-00663-9

**Published:** 2020-09-01

**Authors:** Martina Lang, Marlene Pröschel, Nico Brüggen, Uwe Sonnewald

**Affiliations:** Division of Biochemistry, Department of Biology, Friedrich-Alexander-University Erlangen-Nuremberg, Staudtstrasse 5, 91058 Erlangen, Germany

**Keywords:** SpyTag/SpyCatcher, Intermolecular isopeptide bonding, *Nicotiana benthamiana*, Magnetic beads, One-step organelle purification, Chloroplasts, Mitochondria

## Abstract

**Background:**

Up-to-now, several biochemical methods have been developed to allow specific organelle isolation from plant tissues. These procedures are often time consuming, require substantial amounts of plant material, have low yield or do not result in pure organelle fractions. Moreover, barely a protocol allows rapid and flexible isolation of different subcellular compartments. The recently published SpySystem enables the in vitro and in vivo covalent linkage between proteins and protein complexes. Here we describe the use of this system to tag and purify plant organelles.

**Results:**

We developed a simple and specific method to in vivo tag and visualize, as well as isolate organelles of interest from crude plant extracts. This was achieved by expressing the covalent split-isopeptide interaction system, consisting of SpyTag and SpyCatcher, in *Nicotiana benthamiana* leaves. The functionality of the SpySystem in planta, combined with downstream applications, was proven. Using organelle-specific membrane anchor sequences to program the sub-cellular localization of the SpyTag peptide, we could tag the outer envelope of chloroplasts and mitochondria. By co-expression of a cytosolic, soluble eGFP-SpyCatcher fusion protein, we could demonstrate intermolecular isopeptide formation in planta and proper organelle targeting of the SpyTag peptides to the respective organelles. For one-step organelle purification, recombinantly expressed SpyCatcher protein was immobilized on magnetic microbeads via covalent thiol-etherification. To isolate tagged organelles, crude plant filtrates were mixed with SpyCatcher-coated beads which allowed isolation of SpyTag-labelled chloroplasts and mitochondria. The isolated organelles were intact, showed high yield and hardly contaminants and can be subsequently used for further molecular or biochemical analysis.

**Conclusion:**

The SpySystem can be used to in planta label subcellular structures, which enables the one-step purification of organelles from crude plant extracts. The beauty of the system is that it works as a covalent toolbox. Labeling of different organelles with individual tags under control of cell-specific and/or inducible promoter sequences will allow the rapid organelle and cell-type specific purification. Simultaneous labeling of different organelles with specific Tag/Catcher combinations will enable simultaneous isolation of different organelles from one plant extract in future experiments.

## Background

An elegant and innovative way to covalently link proteins and create artificial multiprotein assemblies or team works, is the use of molecular superglue systems based on engineered Ig-like domains [1–5]. The most efficient, best characterized and therefore widely used posttranslational protein coupling reagents, SpyCatcher and SpyTag, can be used for stable, rapid, irreversible and specific linkage of proteins [4, 6, 7]. The connection is stable under a wide range of conditions, including heat, pH, detergents and mechanical forces [3]. The SpySystem has been used for a broad range of applications, including the production of vaccines [8–12], hydrogels [4, 13–16], the functionalization of surfaces and the construction and spatial organization of multiprotein complexes [17–24], among others [25, 26]. SpyCatcher and SpyTag are known to efficiently work in vitro and in vivo, extra- and intracellular, but so far little is known to which extend they are also functional in planta [3, 6, 27, 28]. The aim of our study was to test the applicability of the SpySystem for the development of a programmable and specific organelle labeling and isolation toolbox. The covalent character of this system could be advantageous for many already existing applications in plant biotechnology and improve established protocols.

In our study, we demonstrate expression and functionality of the SpySystem in planta. The system was used to label outer membranes of chloroplasts and mitochondria using a two-component system. Moreover, we demonstrate that specific decoration of chloroplasts and mitochondria with the SpyTag peptide fused to organelle-specific outer membrane anchors allows the rapid and one-step purification of both organelles from crude plant extracts.

Standard organelle isolation protocols for plant material are mostly based on differential centrifugation, density gradients or a combination of both [29–31].They are often time consuming, require substantial amounts of fresh plant material and can result in contaminated organelle fractions [32–35]. Affinity-based purification protocols are less time-consuming (< 1 h), consist mostly of the incubation of plant homogenate with a carrier material (e.g. beads) followed by washing steps, and deliver organelles with high purity that can be used directly for subsequent analysis. Several epitope-tagging protocols achieving organelle isolation via co-IP have been published, focusing exclusively on one type of organelle [36–42].

Split-isopeptide Catcher–Tag systems could be used as specific, covalent alternatives to the non-covalent, affinity-based organelle isolation techniques, such as the biotin-streptavidin based isolation of nuclei or mitochondria published as INTACT [37] and IMTACT method [42], the Strep-Tactin based isolation of tagged mitochondria [40], the immunogenic chloroplast isolation via YFP [36], or epitope-tagging approaches for mitochondria isolation [38, 39, 41].

The INTACT and IMPTACT method allow the isolation of nuclei or mitochondria via protein–protein interaction, with the help of biotinylated targeting proteins that bind to streptavidin coated beads when mixed [37, 42]. To achieve biotinylation of targeting fusion constructs, a bacterial biotin ligase needs to be co-expressed. For the isolation method via Strep-Tactin [40, 43], organelles were labeled with a targeting construct including Twin-*Strep*-tag. Affinity purification was achieved by incubating plant or mammalian cell filtrate with Strep-Tactin coated beads followed by washing.

Purification can also be achieved via epitope-tagging of desired organelles with the help of targeting fusion constructs, combined with the immobilization of specific antibodies on magnetic beads. Immunogenic isolation was described for mitochondria via HA-Tag [38, 39, 41], Strep-Tag [40] or for chloroplasts via YFP fused to a targeting sequence [36]. Similar purification methods described for mammalian or other systems are based on GFP [44] or on immobilized antibodies directed against organelle surface proteins e.g. TOM22 [45–47].

With an efficient split-isopeptide system at hand, the covalent character of the SpySystem and the specificity of organelle targeting peptides were combined to create a quick and easy as well as robust method to individually and specifically tag, manipulate and furthermore isolate plant organelles of interest.

The beauty of the system is the broad range of applications and the ability to label different targets and structures, creating a covalent plant manipulation toolbox. Furthermore, various organelles could be labelled in a cell-specific manner by the use of cell-specific promoter sequences. Combining different, orthogonal Tag/Catcher systems like the Spy-, 4oq1- or Snoop-System, [5, 48, 49] in combination with promoters, labeling and isolation of different organelles of interest (such as nuclei, chloroplasts and mitochondria), polysomes and intracellular vesicles (ER-derived, Golgi-derived etc.) at the same time can be seen as a long-term goal. Individually isolated organelles combined with subsequent analysis (proteomics, metabolomics) could deliver a closer insight in cell-type specific metabolism, signaling and other mechanisms [34, 36].

Additionally, the system could also be used for the intracellular organization of metabolons or signalosomes by exchanging the fluorescent protein with enzymes or proteins of interest. Metabolic pathway activities and overall yields can be increased in combining enzymes in one complex. The possibility to construct such efficient multienzyme assemblies in an artificial way was realized with the help of the SpySystem [15, 19, 20, 22–24, 50–52]. In combining multiprotein assemblies with targeting sequences, pathways can be introduced, specifically redirected and immobilized exactly where the desired reaction is needed.

In conclusion, we created a versatile plant engineering toolbox based on the covalent SpySystem. We show specific and individual labeling of several organelles in planta and developed a rapid isolation method for SpyTag-labeled organelles. The system has the potential to become an important tool in plant biotechnology and metabolic engineering and will open a broad spectrum of new applications in planta.

## Results

### Design of SpyTag and SpyCatcher fusion constructs to target and label chloroplasts and mitochondria

**Fig. 1:**
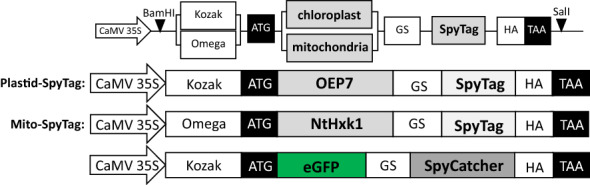
Schematic representation of SpyTag and SpyCatcher fusion constructs for plant organelle targeting in *Nicotiana benthamiana.* All plant expression constructs are under control of the CaMV35S promoter and have a Kozak or Omega sequence in front of the start codon. For SpyTag fusion constructs, the organelle targeting sequence is at the N-terminus followed by a flexible glycine–serine linker (GSGSGSG), the SpyTag sequence and an HA-Tag at the C-terminus. Targeting sequences are OEP7 for chloroplastic (Plastid-SpyTag), and *NtHxk1* for mitochondrial targeting (Mito-SpyTag). As reporter construct, eGFP was C-terminally fused to SpyCatcher, separated by glycine–serine linker and a C-terminal HA-Tag

Metabolome analysis at the sub-cellular level is a challenging task. Most methods for organelle isolation are time demanding and metabolic shifts during preparation cannot be excluded. To circumvent this, non-aqueous fractionation can be applied. This method allows the rapid fractionation of tissues and provides a fairly good oversight of major cellular compartments. The method, however, does not allow separation of all organelles and cell-specific studies in plant tissues are impossible. Given the lack of suitable methods, we wanted to develop a universal system which would allow cell-specific labelling and purification of plant organelles in one-step as a basis for metabolite analysis. As proof of principle, we made use of the well described SpySystem. The system is based on two components, SpyCatcher and SpyTag. Both partners can be fused to proteins or activated surfaces. If SpyTag and SpyCatcher come into close vicinity, a covalent isopeptide bond is autocatalytically formed between them. This intermolecular isopeptide bond formation can be used to design stable protein complexes or to bind proteins to functionalized surfaces. To validate, whether this system could be used for organelle purification, we developed a system in which the SpyTag peptide is targeted to the surface of mitochondria or chloroplasts. Both targeting fusion constructs consist of a specific organelle targeting sequence at the N-terminus followed by SpyTag and an HA-Tag at the C-terminus (Fig. 1). The domains are separated by a flexible 7× glycine–serine linker (GS) to allow proper protein folding and facilitate the accessibility of both, the Catcher to bind to the Tag and the targeting sequence to be directed to and integrated at the final location.

The targeting sequences were selected according to their reported function and properties. For chloroplast targeting, we chose the anchor peptide OEP7 (*outer envelope membrane protein 7*) which is known to have chloroplast targeting properties. The N-terminus of OEP7 is integrated in the membrane, the C-terminus is exposed to the cytosolic leaflet of the outer envelope [53, 54]. Therefore, C-terminal OEP7-SpyTag fusion constructs (Chloroplast-SpyTag: Plastid-SpyTag, Fig. 1) were made to ensure the accessibility of SpyTag from the cytosol.

Additionally, we chose to target mitochondria as they are essential for metabolism and energy production and by that an important subject for metabolomics and proteomics [29, 32, 55]. For mitochondrial targeting, the first 42 N-terminal amino acids of hexokinase 1 from *Nicotiana tabacum* (*NtHxk1*) were fused to SpyTag (Mitochondria-SpyTag: Mito-SpyTag, Fig. 1.). Previously it was shown that *NtHxk1* can direct GFP to the outer mitochondrial envelope [56].

To validate the accessibility of the SpyTag domain, a cytosolic and soluble eGFP-SpyCatcher fusion construct was designed. To achieve this, eGFP was fused to the N-terminus of SpyCatcher. Both domains were separated by a flexible glycine-serine linker (eGFP-SpyCatcher, Fig. 1).

### Expression of organelle-specific SpyTag peptides and verification of intermolecular isopeptide bond formation by co-expression of eGFP-SpyCatcher in leaves of *Nicotiana benthamiana*

**Fig. 2:**
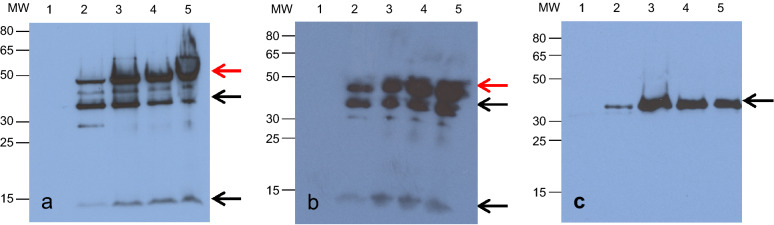
Transient expression kinetics and covalent intermolecular bond formation between organelle-specific SpyTag and eGFP-SpyCatcher in *N. benthamiana*. Western Blot analysis of leaf samples transiently co-expressing HA-tagged organelle-specific SpyTag and eGFP-SpyCatcher. Samples were taken every 24 h until 96 h after infiltration (lane 1–5). Lane 1: 0 h, lane 2: 24 h, lane 3: 48 h, lane 4: 72 h and lane 5: 96 h after infiltration. Co-expression and interaction of eGFP-SpyCatcher and **a** Plastid-SpyTag (plastid-specific; 10 kDa) and **b** Mito-SpyTag (mitochondrion-specific; 8 kDa) is shown. **c** The expression of eGFP-SpyCatcher alone (38 kDa, negative control). MW stands for molecular weight (kDa). Black arrows indicate the single protein bands, red arrows show the covalent product after SpyTag/SpyCatcher interaction

To test expression and sub-cellular localization of both organelle-specific SpyTag-fusion proteins, a transient co-expression experiment in *Nicotiana benthamiana* was conducted. To this end, expression vectors encoding the individual organelle-specific SpyTag proteins or eGFP-SpyCatcher (described in Fig. 1) were transformed into *Agrobacterium tumefaciens*. Subsequently, transformed agrobacteria were mixed or individually infiltrated into leaves of *N. benthamiana*. Following infiltration, expression of SpyTag and SpyCatcher fusion proteins was followed by immunoblotting over a period of 96 h. Detection of the fusion proteins was achieved by the use of an anti-HA specific antibody. This analysis revealed stable accumulation of the fusion proteins if SpyTag (Additional file 1: Fig. S1), or eGFP-SpyCatcher (Fig. 2) were expressed individually or in combination (Fig. 2). As evident (Fig. 2), co-expression of SpyTag and SpyCatcher fusion proteins not only resulted in accumulation of the individual fusion proteins but also in the accumulation of a higher molecular weight protein which resembles the interaction between both proteins (Fig. 2, red arrow). This higher molecular weight protein band was absent when SpyTag (Additional file 1: Fig. S1) or SpyCatcher (Fig. 2) were expressed individually. The higher molecular weight band accumulated 24 h to 48 h after infiltration, depending on the expression of both educts. When comparing the ratio of interacted products and unreacted educts, it can be noticed that in most cases more product can be found than free educts. If both, Tag and Catcher constructs, are available in an equimolar ratio the efficiency of the interaction is very high even when the proteins are expressed in a complex environment [3]. For an interaction to take place, both constructs have to be available and functional in the same cell which cannot always be achieved when co-expressed from independent agrobacteria strains.

### Microscopic visualization of organelle targeting of SpyTag by co-expression of eGFP-SpyCatcher

**Fig. 3:**
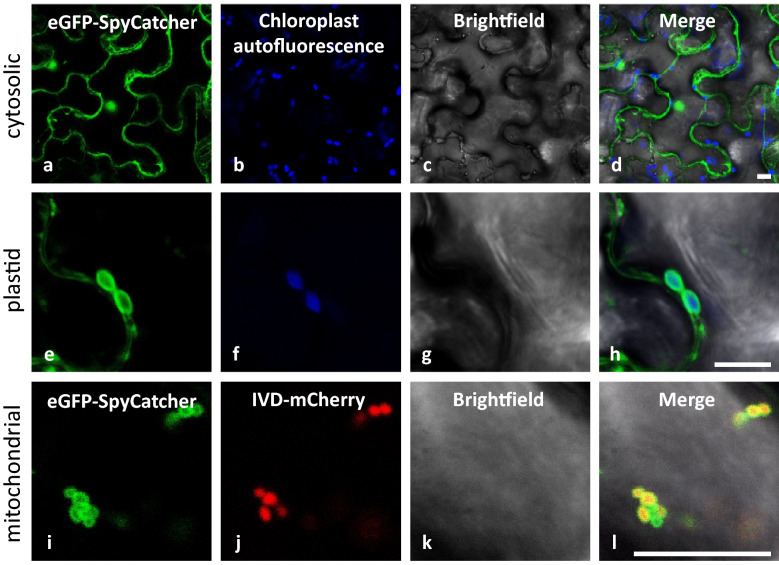
Validation of the subcellular localization by covalent interaction in planta between co-expressed organelle-specific SpyTag and eGFP-SpyCatcher. Organelle-specific SpyTag constructs and eGFP-SpyCatcher were transiently co-expressed in leaves of *Nicotiana benthamiana* plants. CLSM analysis was performed 72 h/96 h after infiltration. Expression of eGFP-SpyCatcher alone revealed cytosolic and nuclear localization (**a**–**d**). When co-expressed with organelle-specific SpyTag constructs, eGFP fluorescence is directed towards the targeted organelles. Chloroplasts were targeted by co-expressing Plastid-SpyTag (**e**–**h**), and mitochondria by co-expressing Mito-SpyTag (**e**–**h**). Autofluorescence of chloroplasts is shown in blue (**a**–**h**), the mitochondria marker IVD-mcherry is shown in red (**i**–**l**). Scale bars represent 10 µm

After having shown that SpyTag and SpyCatcher fusion proteins can interact in planta and form stable isopeptide bonds (Fig. 2), correct sub-cellular localization of the fusion proteins was validated by confocal laser scanning microscopy (CLSM). To this end, eGFP-SpyCatcher was either expressed alone, or in combination with chloroplast (Plastid-SpyTag) or mitochondrion specific SpyTag (Mito-SpyTag). Chimeric genes were transiently expressed in *N. benthamiana* leaves by agrobacterium infiltration. 72 h (in case of soluble GFP and chloroplast targeting) or 96 h (in case of mitochondrion targeting) after inoculation, leaf samples were taken and inspected by CLSM. As expected, expression of eGFP-SpyCatcher, in the absence of SpyTag peptides, resulted in cytosolic and nuclear localization of GFP (Fig. 3). Unspecific nuclear localization is commonly observed for cytosolic GFP fusion proteins [57]. Transient expression of free eGFP under control of the CaMV35S promoter alone or in combination with the organelle-specific SpyTag peptides (Additional file 2: Fig. S2) confirmed these findings. Co-expression of soluble GFP (lacking the SpyCatcher peptide) with SpyTag did not result in altered GFP localization, demonstrating that eGFP does not un-specifically interact with the SpyTag peptide. When eGFP-SpyCatcher was co-expressed with Plastid-SpyTag, the localization of GFP fluorescence changed and showed chloroplast localization (Fig. 3). More precisely, the eGFP signal was localized at the surface of chloroplast and surrounds the chlorophyll autofluorescence signal (Fig. 3). It can be observed that residual unreacted eGFP-SpyCatcher remains in the cytosol which can be explained by different expression levels and as follows, interaction efficiency and accumulation of educts also shown by Western Blot analysis (Fig. 2). For mitochondrion targeting, *NtHxk1* was used as targeting sequence in Mito-SpyTag. To visualize mitochondria, a mitochondrial marker protein (IVD-mCherry) was included in the transient expression of Mito-SpyTag and eGFP-SpyCatcher. IVD-mCherry consists of the mitochondrial transit peptide of isovaleryl dehydrogenase fused to the N-terminus of mCherry, resulting in the import of mCherry into mitochondria. Co-expression of Mito-SpyTag and eGFP-SpyCatcher, in combination with the mitochondrial marker IVD-mCherry, showed a clear colocalization of the GFP and mCherry. The eGFP signal, directed to the outer envelope membrane, surrounded the mCherry signal of the marker (IVD-mCherry) inside the mitochondria (Fig. 3).

### Functionalization of magnetic maleimide-beads with recombinant cysteine-SpyCatcher

Based on immunoblot analysis, we could demonstrate intermolecular isopeptide bond formation in planta. By CLSM we additionally verified that the chosen targeting sequences allowed organelle-specific localization of the SpyTag peptide. Next, we wanted to use the specific targeting of SpyTag for organelle purification. To allow purification of SpyTag decorated plant organelles, we developed a simple protocol for the cross-linking of recombinant SpyCatcher to maleimide activated magnetic beads. The maleimide group specifically reacts with the sulfhydryl group of cysteine residues, forming a stable thioether linkage. Naturally, the SpyCatcher protein does not contain any cysteine residue. Therefore, we introduced one cysteine residue, separated by a TEV cleavage site, at the N-terminus of SpyCatcher by PCR. The modified SpyCatcher sequence was subsequently introduced into the bacterial expression vector pQE-9, adding six N-terminal histidine residues for affinity purification to the fusion protein (see “Methods”). Recombinant SpyCatcher proteins were produced in *E. coli* and purified via Ni-NTA affinity chromatography (for details see “Methods”). For covalent linkage, purified Cysteine-SpyCatcher protein (Cys-SpyCatcher) was incubated with maleimide activated magnetic beads. Loading efficiency and functionality of Cys-SpyCatcher were tested by incubating SpyCatcher-beads with recombinant and purified eGFP-SpyTag (Additional file 3: Fig. S3). As negative control unloaded maleimide beads were incubated with eGFP-SpyTag. Only Cys-SpyCatcher loaded beads mixed with eGFP-SpyTag protein showed a strong GFP signal at the surface of the magnetic beads when observed under a fluorescence microscope (Additional file 3: Fig. S3). This indicates successful loading of maleimide beads with Cys-SpyCatcher protein and verified the functionality of the immobilized proteins.

### Specific Tag/Catcher mediated isolation of Plastid-SpyTag labeled chloroplasts

**Fig. 4:**
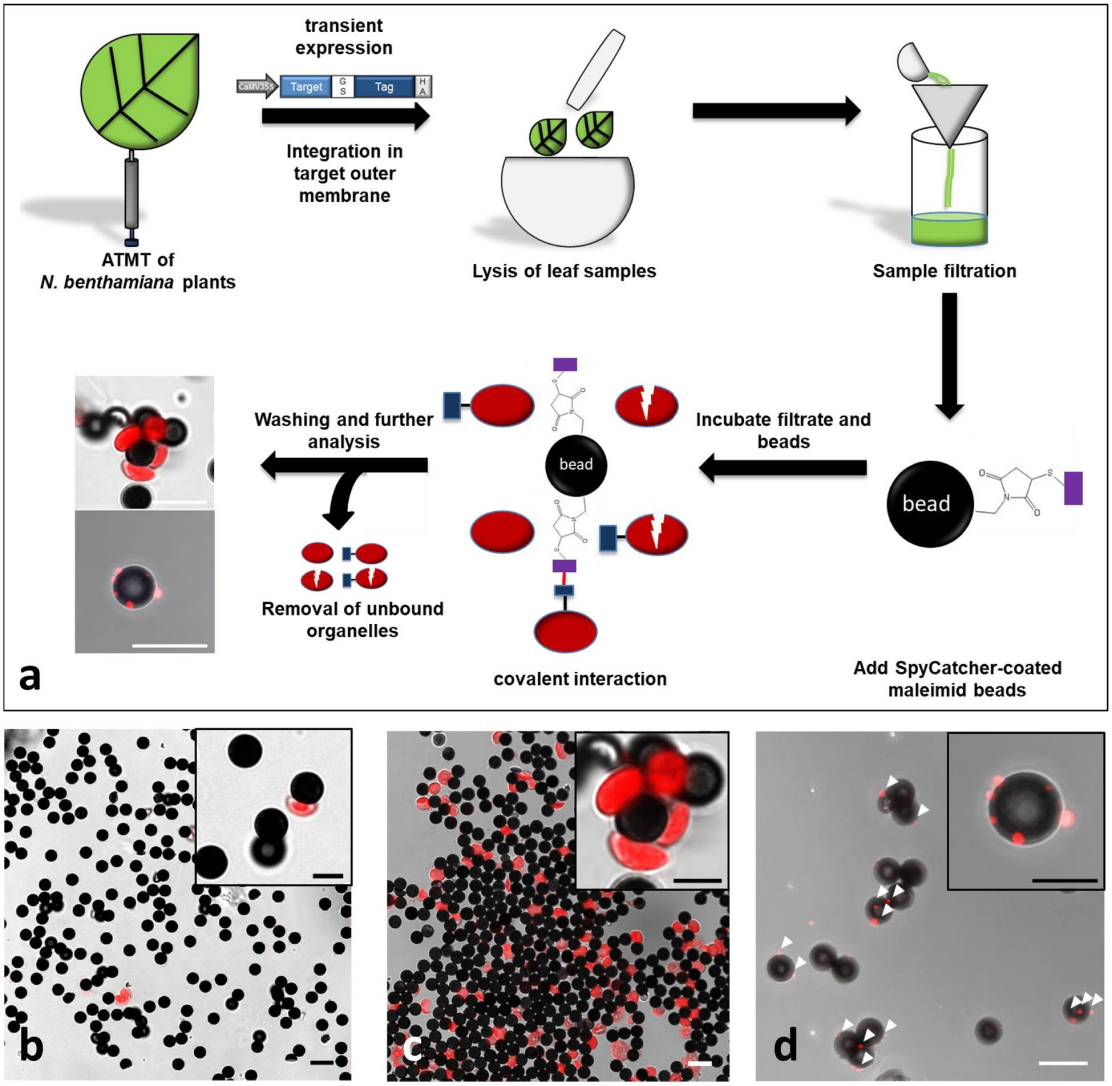
Workflow and CLSM analysis of purified SpyTag-labelled organelles isolated with magnetic SpyCatcher-beads via SpyTag/SpyCatcher interaction. **a** Transiently transformed tobacco leaves expressing organelle-specific SpyTag fusion proteins were harvested homogenized, filtered through miracloth and mixed with SpyCatcher-coated magnetic beads. After incubation, beads with covalently bound organelles were washed and further analysed. **b** Washed beads after incubation with wild type filtrate (negative control). **c** Washed beads after incubation with filtrate from plants expressing Plastid-SpyTag show co-localization of beads (dark dots) and chloroplasts (chloroplast autofluorescence is shown in red). **d** Washed beads after incubation with filtrate from plants expressing Mito-SpyTag, show co-localization of beads (dark dots) and mitochondria (red) indicated by white arrows. Mitochondria were stained with MitoTracker Orange CM-H2TMRos. Scale bars represent 10 μm and 5 µm in the zoomed images

**Fig. 5:**
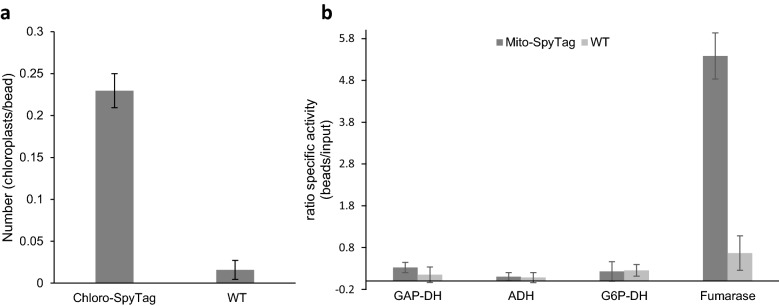
Quantification of isolated chloroplasts and mitochondria bound to SpyCatcher beads after purification. **a** Quantification of isolated chloroplasts from plants transiently expressing Plastid-SpyTag or wildtype plants. Chloroplasts in direct proximity to beads were counted based on representative microscopic images and the ratio of chloroplasts per bead was calculated. **b** The enrichment of mitochondria from plants transiently expressing Mito-SpyTag or wildtype plants was determined by marker enzyme assays. The ratio of the specific activity from different marker enzymes of the input fraction (crude plant filtrate before incubation with beads) and the bead fraction (beads after incubation with plant filtrate and washing steps) was calculated. GAP-DH: NADP-dependent glyceraldehyde-3-phosphate dehydrogenase, chloroplast marker; ADH: alcohol deydrogenase, cytosol marker; G6P-DH: NADP-dependent glucose-6-phosphate dehydrogenase, cytosol marker; Fumarase, mitochondria marker)

After having shown that Cys-SpyCatcher loaded beads can covalently bind eGFP-SpyTag, the functionalized beads were used to purify SpyTag decorated chloroplasts. To test the applicability of the system, we transiently expressed Plastid-SpyTag in leaves of *N. benthamiana*. 72 h after inoculation, leaf samples (~ 0.5 g) of Plastid-SpyTag expressing plants and control plants (without transient expression of Plastid-SpyTag) were taken and immediately grinded with isolation buffer in a prechilled mortar (see “Methods”). The homogenate was filtered through miracloth before mixing with SpyCatcher-coated magnetic beads. After 30 min incubation at 4 °C beads were washed and diluted in appropriate buffer (Fig. 4a) for further analysis. When control leaf extracts were incubated with SpyCatcher-beads, there was no evidence for unspecific interaction between beads and chloroplasts. Magnetic particles (dark dots) and red chloroplast autofluorescence did rarely colocalize (Fig. 4b). However, when filtrates of Plastid-SpyTag expressing plants were incubated with SpyCatcher loaded magnetic beads, a clear localization of chloroplasts and beads in close proximity could be observed under the microscope, which proves the interaction of SpyCatcher-coated beads and SpyTag-labeled chloroplasts (Fig. 4c). Quantitative analysis additionally confirmed the visual impression and showed that significantly more chloroplasts were isolated when Plastid-SpyTag leaf filtrate was incubated with SpyCatcher-coated beads compared to control extracts (Fig. 5). Quantification revealed that on average 4–5 beads are needed to isolate one chloroplast (22.8% of beads carry a chloroplast). Beads incubated with wild type extract show a minor contamination with unspecifically co-isolated chloroplasts (1.4% of beads have an unbound chloroplast in their proximity).

### Specific SpyCatcher–SpyTag mediated mitochondria isolation

Next, we wanted to show that beside chloroplasts also Mito-SpyTag labelled mitochondria can be specifically isolated from crude leaf extracts. Similar to the procedure described for chloroplasts, Mito-SpyTag was transiently expressed in leaves of *N. benthamiana* plants. For the isolation procedure, filtrates of tobacco plants expressing Mito-SpyTag were mixed and incubated with SpyCatcher-coated magnetic beads (Fig. 4a) whereas control extracts served as a negative control. To allow microscopic detection of mitochondria, plant extracts were mixed with the dye MitoTracker Orange CM-H2TMRos. The dye is only taken up by intact mitochondria showing an active membrane potential and results in fluorescent labeling. After washing, a clear localization of fluorescent MitoTracker Orange and beads in direct proximity could be observed under the microscope, when beads were incubated with Mito-SpyTag transformed plant extracts (Fig. 4d), showing the interaction of SpyCatcher-beads and SpyTag-labeled mitochondria. However, when control leaf extracts, combined with MitoTracker Orange, were incubated with SpyCatcher-beads, there was no evidence for interaction between beads and mitochondria. The magnetic particles (dark dots) and the red fluorescent of MitoTracker Orange labeled mitochondria did rarely colocalize (Fig. 4b).

**Fig. 6:**
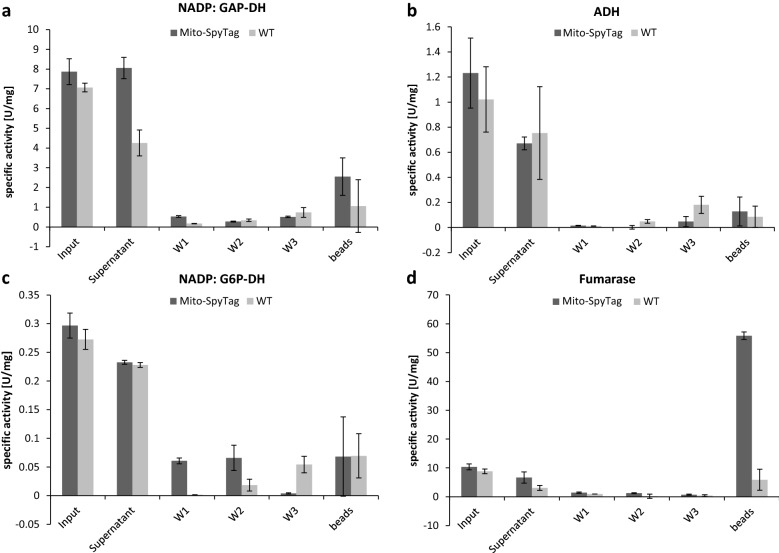
Marker enzyme assays of different fractions during mitochondria isolation. Enzyme activity of plastidic, cytosolic and mitochondrial marker enzymes measured in the following fractions: Input (plant filtrate before incubation with SpyCatcher beads), Supernatant (plant filtrate after incubation with SpyCatcher beads, W1–W3 (Washing fractions) and beads (beads with isolated organelles). **a** Shows specific NADP-dependent glyceraldehyde-3-phosphate dehydrogenase activity (NADP: GAP-DH; plastidic marker), **b** shows specific alcohol dehydrogenase activity (ADH; cytosolic marker), **c** shows specific NADP-dependent glucose-6-phosphate dehydrogenase activity (cytosolic marker) and **d** shows specific fumarase activity as a marker for mitochondria enrichment

Additionally, quantitative and qualitative analysis was performed to further characterize the enrichment, purity and intactness of mitochondria isolated in the bead fraction. Quantitative analysis was performed as described for chloroplast. Counting revealed that 31.1% of all beads carry a bound mitochondrion. The enrichment was also determined by measuring the mitochondrial fumarase activity. Quantification of the enzyme activity revealed a 5.4-fold enrichment of fumarase activity in the bead fraction compared to the crude input fraction (Fig. 5) (Input fraction: filtrated plant extract before mixing with SpyCatcher-coated beads). Furthermore, the purity of the isolated mitochondria and possible contaminations by cytosolic or chloroplastic proteins, was analyzed. To do so, activities of cytosolic marker enzymes [alcohol dehydrogenase (ADH), NADP-dependent Glucose-6-Phosphate dehydrogenase (NADP:G6P-DH)] and chloroplastic marker enzymes [NADP-dependent glyceraldehyde-3-Phosphate dehydrogenase (NADP:GAP-DH)] were measured at different timepoints during the isolation procedure (Fig. 6). Analysis revealed a slight contamination with chloroplast proteins (Fig. 6a), which was consistent with microscopic analysis, but cytosolic contaminations in the bead fraction were barely measurable (Fig. 6b, c). Incubation of SpyCatcher-coated beads with control extracts served as a negative control and no enrichment of mitochondria or other compartments could be measured. This finding confirmed the specificity of the interaction between SpyCatcher-beads and SpyTag-labeled organelles. It could be shown that intact mitochondria can be isolated from crude plant extracts by transiently labeling the organelles with SpyTag and incubating the plant filtrate with SpyCatcher-coated beads.

In conclusion, the labeling of plant organelles with SpyTag by transient expression of targeting SpyTag fusion constructs, enables the specific isolation of desired organelles with only the help of SpyCatcher-coated magnetic beads. The procedure is rapid, flexible and easy, can be performed without the need for expensive equipment and only a little amount of transgenic plant material and beads is needed to achieve sufficient yield. The method can be transferred to different organelles and structures, considering the exchangeability of the targeting sequence. Additionally, the protocol can be modified depending on the downstream application to include a centrifugation step or decreased incubation length.

## Discussion

In our study we could show that the SpySystem, the molecular superglue derived from gram-positive bacteria [2–4, 58], is functional in planta. SpyCatcher and SpyTag fusion proteins find each other in the complex environment of the plant cell and are able to form a covalent isopeptide bond [58]. These findings open a whole new era of plant biotechnological applications where stable, post-translational interactions are advantageous and needed, shown for example in the construction of enzyme complexes [28]. One major advantage of covalent protein interaction systems is the irreversible and stable connection between Tag and Catcher under all kinds of conditions [3] allowing applications in areas, where noncovalent interaction systems cannot function properly, e.g. extreme pH, detergents or mechanical forces [3, 6]. In contrast to non-covalent or affinity-based interaction systems, the SpySystem shows no unwanted release of coupled partners caused by dissociation because of the stability of the covalent isopeptide bond.

The fusion of SpyTag to organelle-specific targeting sequences allows decoration of the organelle surface. Considering the proper folding of the membrane anchor, the SpyTag peptide can be accessible in the cytosol, as demonstrated by the specific binding of soluble, cytosolic eGFP-SpyCatcher to chloroplasts and mitochondria. By exchanging the organelle-specific targeting sequence, most subcellular structure, e.g. other organelles, vesicles, membranes, or the cytoskeleton can be addressed [59]. Furthermore, the system could be used to immobilize proteins of interest at a distinct site in the cellular space and control their place of action, for example by coupling suitable enzymes to organelles. Molecular superglues can be a useful tool for metabolic engineering, which allows to redirect the metabolic flux inside the cell, introduce new, spatially controlled pathways or design metabolic networks from scratch by creating artificial multienzyme complexes [6, 20]. The size of the SpyTag peptide is beneficial for protein fusions as it only consists of 13 amino acids [3] resembling the molecular weight of commonly used epitope tags [60] e.g. HA-Tag (9 aa), Flag-Tag (8 aa) or myc-Tag (10 aa). The bigger SpyCatcher protein could be a disadvantage considering protein fusions. With a molecular mass of 116 amino acids [1, 61] fusions of SpyCatcher could influence the function of the partner it is fused to. Expression, stability and folding of fusion proteins can be negatively influenced, resulting in a loss of function, for example in enzymes or proteins, where the fusion could also affect the formation of multimers by steric hindrance. Nevertheless, those problems could be tackled and solved with the help of bioinformatics and molecular modelling. With this at hand, the SpyTag fusion site (N-, C-terminally or internally) [3] as well as the fusion site of SpyCatcher (N- or C-terminally) [4] can be adjusted according to the protein structure and stability or the length of flexible linkers can be optimized to increase the accessibility.

In our study, we created a rapid and simple isolation protocol for intact, SpyTag-labeled organelles from crude leaf filtrates with the help of SpyCatcher-coated magnetic beads. Our protocol is similar to affinity-based isolation methods and helps to overcome the general limitations of standard isolation protocols as density gradients, like the need of substantial amounts of starting material, long centrifugation steps, low yield and a protocol length of several hours. The isolation procedure is based on the specific interaction of SpyTag and -Catcher and needs no further treatment like additional centrifugation steps. Nevertheless, short centrifugation of plant extracts before incubation with SpyCatcher-beads could on the one hand minimize contamination from other types of organelles and cell debris but on the other hand have an impact on organelle integrity and metabolite composition. The complete isolation procedure only takes from minutes up to < 1 h, depending on the incubation time of plant filtrate and SpyCatcher-beads. SpyCatcher and SpyTag are known to form isopeptide bonds very quickly in a few minutes [3], so incubation length can be adjusted according to yield, integrity or quality of organelles, depending on the desired downstream application. The little amount of plant material needed for organelle isolation can be very beneficial e.g. when working with seedlings, mutants or generally small plants. In our study 0.5 g of transiently transformed leaf material (fresh weight) was sufficient for chloroplast and mitochondria isolation in combination with 50 µl SpyCatcher-beads. Efficiency and yield might be increased by extending the incubation time as Catcher/Tag interaction is irreversible and dissociation is not an issue. However, prolonged incubation time could have a negative effect on organelle intactness and activity as well as metabolite composition. For sensitive applications like metabolomics, incubation should be as short as possible. Higher yields of isolated organelles could also be achieved by upscaling (varying bead to plant filtrate ratio by incubating more beads with plant filtrate) and by using transgenic plants that stably express the targeting SpyTag constructs. The organelle purification protocol described here allowed the isolation of intact chloroplasts and mitochondria in sufficient amounts for further analysis as yield was favoured. Microscopic analysis revealed hardly unspecific binding of chloroplasts to beads when one of the reactive partners (Tag or Catcher) was missing (Fig. 4b). Un-specifically co-isolated chloroplasts are a commonly observed issue when purifying organelles from green plant tissues. To minimize this effect, we used LoBind Eppendorf tubes during the incubation of beads and plant filtrate. The number of unwanted organelles, cell debris and other contaminants in the organelle fraction could additionally be reduced by adding a centrifugation step prior to bead incubation.

For the creation of an organelle isolation toolbox, we exchanged the targeting sequence to target and isolate mitochondria following the same protocol described for chloroplast isolation.

Isolated mitochondria bound to SpyCatcher-beads after incubation seemed intact and active, tested via fumarase activity measurements and MitoTracker staining. MitoTracker Orange CM-H2TMRos is only fluorescent after oxidation and accumulates in mitochondria with an active respiratory chain [62]. Comparison of marker enzyme activities measured in different fractions during the isolation, resulted in a ~ 5.4 fold enrichment of mitochondrial fumarase activity in the bead fraction when compared to the activity of the input fraction, where tagged and untagged mitochondria are present as well as a multitude of different proteins. Marker enzyme activity measurements revealed a small contamination of chloroplasts in the bead fraction which is consistent with microscopic analyses and findings from previous chloroplast isolations. As mentioned above, contamination by chloroplasts or other organelles could be minimized by adding a centrifugation step prior to incubation of plant filtrate with SpyCatcher-beads.

Prior to measuring the activity of isolated mitochondria, we sonicated the bead fraction for lysis of all bound organelles for further analysis. If intact organelles are required, a TEV cleavage site located on the SpyCatcher constructs allows separation of the Tag/Catcher interaction product from the beads, bypassing the covalent interaction. Alternatively, a similar cleavage site could be introduced to the targeting SpyTag construct to cut off excess protein residue from the organelles. When intact organelles are not needed, simple lysis and removal of magnetic beads will eliminate most of the interacted products, too, as SpyTag/SpyCatcher are covalently linked to the beads.

Several plant organelle isolation protocols were published in the last years, mostly facilitating the isolation of one type of organelle via affinity-based purification methods [36, 37, 39–42]. In these protocols, either epitope or protein Tags (e.g. HA-, Strep-Tag or YFP) in combination with antibodies or protein-protein interactions (e.g. Strep-Tactin) were used to immobilize labelled organelles on a carrier material like magnetic beads.

Epitope tags are known to provide good recognition and binding abilities towards the respective antibody or protein. Additionally, the epitope Tags used in purification techniques, HA- and Strep-Tag, exhibit a very small size, consisting only of a few amino acids. The addition of such small tags does not add new biological functions and in most cases fusion proteins will retain normal structure and function [39, 63]. But in most of the reported isolation protocols the Tags are not only fused to a targeting sequence for organelle labelling but also to a fluorescent protein (GFP) to allow the localization and visualization of the constructs [37, 39–42]. Nevertheless, antibodies are expensive and as above-mentioned, affinity-based non-covalent systems have limitations in their field of application. The covalent character of the SpySystem could be beneficial compared to affinity-based isolation procedures as isolation and downstream applications under harsh conditions are possible, as long as the organelles, proteins or metabolites survive the treatment.

All steps of the isolation procedure can be done in one tube without the need of expensive materials or devices. The transformation of plants to express the targeting SpyTag construct, the expression and loading of maleimide beads with SpyCatcher protein, as well as the isolation procedure itself can be automated and adjusted to individual needs and downstream applications. The incubation length can be reduced to minimize metabolite changes and enhance integrity, the amount of plant material and/or beads can be increased for higher yield and centrifugation of the plant extract prior to bead incubation could reduce contamination. Additionally the buffer composition could be altered as the SpySystem is functional under a broad range of conditions..Plant transformation can either be performed in a quick transient manner or stably expressing plants can be created, for example with targeting tag constructs under tissue-specific and/or inducible promoter. Expression of targeting Tag constructs under a tissue-specific promoter allows the defined isolation of organelles from a distinct plant tissue or certain cell types. This could be a powerful tool in sample preparation and facilitate the tissue specific analysis of proteome/metabolome data. To express the organelle-specific SpyTag in planta transgenic lines are required limiting the application of the SpySystem to certain model species that can be transformed in a transient or stable manner. The need to genetically modify and express targeting proteins in order to isolate labelled organelles is a general disadvantage of all affinity isolation methods. Isolation via density gradient would allow the use of nontransgenic plant material but exhibits different disadvantages. Maleimide-beads can be stored after coating with SpyCatcher over months in appropriate buffer until needed while retaining their functionality. The protocol is customizable to focus on yield, purity, integrity and downstream applications as described above by adding additional steps like centrifugation or washing or varying the length of incubation. Thus, making the isolation procedure cheaper, quicker, more feasible and easily adjustable to different organelles and needs when compared to existing isolation protocols.

## Conclusions

In our study we confirmed the functionality of the Spy interaction system under covalent isopeptide bond formation in planta and presented several novel applications for subcellular targeting and organelle isolation as a versatile tool for plant metabolic engineering and biotechnology. We showed the expression of SpyTag and SpyCatcher fusion constructs in *N. benthamiana,* enabling the individual targeting and labelling of chloroplasts, and mitochondria by combining the efficiency of the covalent SpySystem with the specificity of organelle targeting peptides. Thereby, a quick and easy as well as robust method to individually and specifically tag, visualize, manipulate and furthermore isolate plant organelles of interest was created. We achieved the specific purification of SpyTag-labelled chloroplasts and mitochondria from crude plant filtrate simply by incubation with SpyCatcher-coated magnetic beads and without the need for further treatment like centrifugation steps. The isolation procedure allows rapid (< 1 h) and specific purification from little amounts of plant material, resulting in intact organelles which can be directly used for subsequent analysis, e.g. metabolomics or proteomics. Split-isopeptide Catcher/Tag systems present a covalent, specific and robust alternative to the available non-covalent, affinity-based organelle isolation techniques. Moreover, the labelling with SpyTag can be combined with tissue-specific promoters allowing the isolation of organelles only from a certain cell type but further studies are required. Individually isolated organelles combined with subsequent analysis could deliver a closer insight in cell-type specific metabolism, signaling and other mechanism [34, 36]. As several Catcher/Tag systems are available by now, the labelling by different organelle-specific Tags, and isolation of multiple organelles from the same extract with respective Catcher-beads can be seen as a long-term goal but functionality of other systems in planta has to be tested.

## Methods

### Cloning of gene constructs

#### pRB-35S plant expression constructs

All constructs used for organelle targeting consist of a specific targeting sequence C-terminally fused to the SpyTag-peptide encoding sequence, separated by a sevenfold GS-linker (GSGSGSG). Sequences of the constructs are provided in Additional file 4: S3. The chloroplast anchor-tag fusion construct Plastid-SpyTag consists of the SpyTag-peptide encoding sequence N-terminally fused to the OEP7 chloroplast outer membrane anchor [53, 54] (Sequence derived from *Arabidopsis thaliana* gene At3g52420; Codon optimized based on the small subunit of potato RuBisCO). To specifically target the outer mitochondrial membrane with Mito-SpyTag, a part of the N-terminal sequence of hexokinase1 from *Nicotiana tabacum* (*NtHxk1*; first 44 amino acids) was used according to Giese [56]. For the reporter-SpyCatcher fusion construct, the SpyCatcher sequence was C-terminally fused to eGFP [64], separated by a glycine-serine linker (GSGSGSG).

Gene constructs for transient expression in *N. benthamiana* were cloned using PCR (standard PCR and overlap-extension PCR) with appropriate primers and DNA-templates or ordered as synthetic genes. All constructs contain BamHI/ SalI restriction sites for directed cloning into the pRB-35S binary plant expression vector (T-DNA, LB, RB, CaMV 35S promoter) and a Kozak sequence (AACA) or Omega sequence for enhancing the translation, followed by a start codon (ATG). All plant expression constructs also contain a C-terminal Hemagglutinin (HA)-tag for Western Blot analysis just in front of the stop codon.

For Plastid-SpyTag (OEP7-SpyTag) and eGFP-SpyCatcher High-fidelity Phusion-DNA Polymerase (Thermo Fisher Scientific) was used for amplification, while Mito-SpyTag (NtHxk1-SpyTag) was ordered as synthetic gene string (GeneArt, Thermo Fisher Scientific). Cloning scheme and primers are listed in Additional file 5: S1 and Additional file 6: S2. Gene constructs were analyzed by agarose gel electrophoresis (1× TBE running buffer) and extracted the from gels (QIAquick Gel Extraction Kit, Qiagen) for subsequent cloning. For sequence verification, synthetic DNA fragments and purified PCR products were ligated into the pCRBlunt vector (T4 DNA Ligase, Thermo Fisher Scientific) and subjected to DNA sequencing.

Double digestion of positive clones and subsequent purification of inserts (QIAquick PCR Purification Kit, Qiagen) was accompanied by T4 DNA ligation into the BamHI/SalI digested pRB-35S vector. Constructs were transformed into chemically competent *Agrobacterium tumefaciens* cells strain C58C1 for subsequent *N. benthamiana* transformation.

#### pQE-9 bacterial expression construct

Recombinant SpyCatcher protein is needed for the functionalization of the bead surface via thioether bond formation between a single cysteine residue artificially introduced to the N-terminus of SpyCatcher and maleimide residues on the beads. The corresponding gene construct was generated via standard PCR with the SpyCatcher sequence as template and appropriate primers (see Additional file 5: S1 and Additional file 6: S2) introducing the nucleotides encoding i.a. for the cysteine residue and a TEV-protease cleavage site.

For testing the efficiency of bead loading after incubation with Cys-SpyCatcher protein, loaded beads were incubated with purified eGFP-SpyTag protein to allow visualization of the Tag/Catcher interaction.

eGFP-SpyTag was generated via PCR using appropriate primers (see Additional file 5: S1 and Additional file 6: S2) and consists of the nucleotide sequence of eGFP, C-terminally fused to the SpyTag-peptide encoding sequence, separated by a GS-linker (GSGSGSG).

The constructs contain restriction sites (Cystein-SpyCatcher: BamHI/ SalI and eGFP-SpyTag: BamHI/PstI) for directed cloning into the IPTG-inducible bacterial expression vector pQE-9. In front of the multiple cloning site, the vector has a start codon followed by the sequence of a 6× histidine Tag that is fused to the N-terminus of inserts for purification of the overexpressed constructs.

For amplification, the high-fidelity Phusion-DNA Polymerase (Thermo Fisher Scientific) was used. The final PCR products were extracted from agarose gel after gel electrophoresis following the manufacturer´s instructions (QIAquick Gel Extraction Kit, Qiagen). Double digestion was followed by T4 ligation into the inducible, bacterial expression vector pQE-9. Sequences were verified by Sanger sequencing (GATC). The pQE-9 expression construct was subsequently transformed into chemically competent M15 [pREP4] *E. coli* cells (Qiagen) for recombinant protein expression after IPTG induction. The sequences of all constructs are listed in Additional file 4: S3.

### *Agrobacterium tumefaciens* mediated transformation of *Nicotiana benthamiana* leaves

Chemically competent *Agrobacterium tumefaciens* cells strain C58C1 were transformed with pRB-35S expression constructs and plated on YEB agar plates (5 g Bacto-beef extract, 1 g yeast extract, 5 g bacto-trypton, 5 g sucrose, 0.24 g unhydrated MgSO_4_, 15 g Agar per 1 l) containing 50 µg/ml Rifampicin, 200 µg/ml Ampicillin, 20 µg/ml Streptomycin, 50 mg/ml Spectinomycin. Single colonies were picked to inoculate an overnight culture of liquid YEB media Agrobacteria cultures were incubated at 28 °C. Transient expression was achieved by *A. tumefaciens* mediated transformation (ATMT). For infiltration, cells were harvested by centrifugation at 4000 rpm for 20 min, and the resulting cell pellet was resuspended in water to adjust an OD_600_ of 1. For co-infiltration studies, constructs were mixed in an equimolar ratio (1:1). *Nicotiana benthamiana* leaves were pressure infiltrated using a needleless syringe. To minimize post-translational gene silencing the gene silencing suppressor p19 [65] was coinfiltrated.

### Western Blot

To verify the transient expression of SpyCatcher- and SpyTag-fusion constructs and the covalent character of the Catcher–Tag interaction in planta, SDS-PAGE followed by Western Blot analysis was performed. The above-mentioned pRB-35S plant expression constructs all contain C-terminal HA-Tags and were transformed into *N. benthamiana* leaves via *A. tumefaciens* mediated transformation (ATMT). Leaf samples were taken every 24 h with a cork borer (diameter 0.9 cm), frozen in liquid nitrogen and stored at – 80 °C until further treatment. Leaf discs were grinded in 70 µl 4× Laemmli sample buffer (200 mM Tris-HCl pH 6.8; 40% glycerol; 18% β-mercaptoethanol; 8% SDS; 0.01% bromophenol blue) and boiled for 10 min at 95 °C. Samples were separated by SDS-PAGE using 12% Bis-Tris gels with 1× MOPS as running buffer (50 mM MOPS, 50 mM Tris, 1 mM EDTA, 0.01% SDS) and blotted afterwards on a nitrocellulose blotting membrane (GE Healthcare) by semi-dry electroblotting procedure. Free binding sites were blocked with 5% milk powder solved in 1× TBS-T buffer (20 mM Tris, 0.5 M NaCl, 0.1% Tween 20, pH 7.5). For detection, Anti-HA peroxidase linked antibodies (Anti-HA-POD, Roche) were used in 1:500 dilution in 1% milk powder solution in 1× TBS-T. Enhanced chemiluminescence (ECL) reaction of luminol allowed the specific detection of HA-tagged proteins.

### Confocal laser scanning microscopy

The inverse confocal laser scanning microscope Leica TCS SP5 II (AOBS) was used for all microscopy studies (CLSM, Leica Mikrosysteme Vertrieb GmbH, Wetzlar). For subcellular localization analyses, leaf segments of transformed tobacco plants were cut 72 h or 96 h after infiltration and observed under the confocal microscope. To analyze the binding of organelles to SpyCatcher-beads, the beads were diluted in appropriate buffer prior to microscopic analysis. Fluorescence of the reporter construct eGFP-SpyCatcher, the mitochondrial marker IVD-mCherry, the Mitotracker Orange, as well as the chloroplasts’ autofluorescence was excited by an Argon laser (488 nm) and DPSS laser (561 nm). EGFP fluorescence was detected from 496 to 560 nm, mCherry fluorescence from 580 to 650 nm, MitoTracker Orange fluorescence from 570 to 625 nm and the chlorophyll autofluorescence from 680 to 780 nm.

### Heterologous protein expression in *E. coli*

Chemically competent *E. coli* M15 [pREP4] cells (Qiagen) were transformed with the pQE-9 expression vector containing the gene construct of interest (Cys-SpyCatcher or eGFP-SpyTag). Transformed cells were plated on LB agar plates with 200 µg/ml ampicillin and 25 µg/ml kanamycin. Single colonies were picked for inoculating an overnight culture in liquid LB with antibiotics (10 g NaCl, 10 g Bactotrypton, 5 g yeast extract per 1 l; 200 µg/ml ampicillin and 25 µg/ml kanamycin) and incubated over night at 28 °C. Appropriate amounts of the overnight culture were used to inoculate a 1 l expression culture to an optical density (OD_600_) of 0.02 and the best time point of induction was determined (see Additional file 7: Fig. S4). The expression cultures were cultivated at 28 °C and shaking at 180–200 rpm until an OD_600_ of 0.5 was reached. Recombinant protein expression was induced by addition of 0.5 mM IPTG (Roth) for 4 h at 28 °C and shaking at 180–200 rpm. After 4 h, cells were harvested by centrifugation at 5000*g* for 20 min at 4 °C. Cell pellets were frozen at – 20 °C for storage.

### Recombinant protein purification and preparation for size exclusion chromatography

Protein purification of recombinant expressed 6× His tagged proteins was performed via nickel-nitrilotriacetic acid agarose affinity chromatography (Ni-NTA, Qiagen) under native conditions (non-denaturing). Harvested cell pellets were thawed on ice and resuspended in lysis buffer containing 1 mM Pefabloc (Roth) and cOmplete Ultra tablets, EDTA free (Merck) protease inhibitor to minimize protein degradation. Sonification steps were performed on ice for efficient cell lysis (6 10-s bursts with cooling on ice after each burst). Soluble and insoluble components of cell lysate were separated by centrifugation (10,000*g*, 30 min, 4 °C). The supernatant fraction was applied to Ni-NTA agarose resin packed in Polypropylene columns (5 ml, Qiagen). The following purification steps, including binding, washing and elution steps were performed according to the manufacturer’s instructions (QiaExpressionist, 2001, Qiagen). Successful purification of the fusion proteins in shown in Additional file 8: Fig. S5.

The following buffers were used: Lysis buffer (50 mM NaH_2_PO_4_ pH 8.0; 300 mM NaCl; 10 mM imidazole), Washing buffer (50 mM NaH_2_PO_4_ pH 8.0; 300 mM NaCl; 20 mM imidazole), Elution buffer (50 mM NaH_2_PO_4_ pH 8.0; 300 mM NaCl; 250 mM imidazole). Purified proteins were dialyzed in Coupling buffer (100 mM NaH_2_PO_4_ pH 7.2, 150 mM NaCl, 0.5 mM EDTA) with the help of dialysis tubes (Servapor MWCO 12K, Serva).

Concentration of recombinant proteins was determined at 280 nm with a NanoDrop spectrophotometer ND-1000 (Peqlab). Prior to size exclusion chromatography (SEC), the purified, dialyzed Cys-SpyCatcher protein was concentrated up to ~ 20 mg/ml using Amicon Ultra-4 centrifugal filter units (MWCO 3K, Merck). SEC was performed with a Superdex 200 column prep grade 16/60 equilibrated with Coupling buffer (Additional file 9: Fig. S6A). Fractions were analyzed by SDS-PAGE followed by Coomassie staining to visualize the protein content and purity (Additional file 9: Fig. S6B). Fractions of interest were pooled and concentrated using an Amicon Ultra centrifugal filter device (MWCO 3 K) and frozen at – 80 °C in small aliquots for storage.

### Functionalization of magnetic Maleimid beads with Cys-SpyCatcher

Magnetic surface activated maleimide beads (diameter 4.5 µm, Ocean NanoTech) were diluted in coupling buffer (100 mM NaH_2_PO_4_, 150 mM NaCl, pH 7.2) to receive a bead concentration of 20 mg/ml (stock-solution). For the coupling reaction between Cys-SpyCatcher and maleimide, protein solution purified via SEC was added to the beads in excess. Beads and protein solution were incubated overnight at 4 °C while shaking at 800 rpm to allow the formation of a covalent thioester bond. After incubation, the supernatant was removed, beads were washed three times with coupling buffer in a magnetic sample rack (DynaMag™-2 Magnet, Thermo Fisher) and bead concentration was adjusted to the initial 20 mg/ml. Bead loading efficiency was tested by incubating loaded SpyCatcher-beads with purified eGFP-SpyTag protein solution for 1 h at room temperature to allow specific covalent Tag/Catcher interaction. After three washing steps with coupling buffer to remove unspecific bound protein, fluorescence directed to the beads’ surface was analyzed by confocal laser scanning microscopy.

### Purification of SpyTag-tagged chloroplasts

To isolate Plastid-SpyTag (OEP7-SpyTag) labelled chloroplasts with SpyCatcher functionalized maleimide beads, 4 leaf discs (~ 0.5 g fresh weight) of 72 h infiltrated tobacco plants were harvested with a cork borer (diameter 2.5 cm) and immediately grinded in 2.5 ml chloroplast isolation buffer (10 mM HEPES-KOH pH 7.0; 0.33 M sorbitol; 0.4 mM KCl; 40 µM EDTA 1% PVPP; 1 mM Pefabloc; cOmplete Ultra protease inhibitor, EDTA free; 2 mM DTT) with a mortar on ice. Plastid-SpyTag leaf extract was filtered through a layer of Miracloth (Merck). 200 µl leaf filtrate (fourfold excess) was mixed with 50 µl SpyCatcher-beads (stock-solution: 20 mg/ml) and incubated for 30 min at 4 °C with mild shaking. To avoid unspecific protein binding to the test tubes’ surface, LoBind Eppendorf tubes were used (Eppendorf). Wildtype leaf extract served as negative control. After incubation, the beads were washed three times in a magnetic sample rack with chloroplast isolation buffer. Beads were diluted in chloroplast isolation buffer for further analysis.

### Purification of SpyTag-tagged mitochondria

For the isolation of Mito-SpyTag labelled mitochondria with SpyCatcher functionalized maleimide beads, 4 leaf discs (~ 0.5 g fresh weight) of 72 h infiltrated tobacco plants were harvested with a cork borer (diameter 2.5 cm) and immediately grinded in 2.5 ml mitochondria isolation buffer (30 mM MOPS pH 7.5; 0.3 M mannitol; 1 mM EDTA; 1 mM Pefabloc; cOmplete Ultra protease inhibitor, EDTA free; 2 mM DTT [66]) with a mortar on ice. Mito-SpyTag leaf extract was filtered through two layers of Miracloth (Merck). 500 µl leaf filtrate (tenfold excess) was mixed with 50 µl SpyCatcher-beads (stock-solution: 20 mg/ml) in LoBind Eppendorf tubes and incubated for 30 min at 4 °C on a tube rotator with very slow rotation. After incubation, the beads were carefully washed three times in a magnetic sample rack with mitochondria isolation buffer. Beads were diluted in mitochondria isolation buffer for further analysis.

### Quantification of isolated organelles

#### Chloroplasts

To quantify the number of isolated chloroplasts specifically bound to SpyCatcher-beads via Plastid-SpyTag/SpyCatcher interaction, microscope images were taken from SpyCatcher beads incubated either with plant filtrate from wildtype or transiently Plastid-SpyTag expressing plants. The number of beads and chloroplasts in direct proximity as well as unbound chloroplasts and empty beads was counted and the ratio of chloroplasts per bead was calculated.

#### Mitochondria

The enrichment of mitochondria on SpyCatcher beads via Mito-SpyTag/SpyCatcher interaction was determined via marker enzyme activity measurements. As marker enzymes fumarase activity was measured for mitochondrial enrichment, NADP: glyceraldehyde 3-phosphate dehydrogenase activity was measured for chloroplastic contamination, NADP: glucose-6-phosphate dehydrogenase and alcohol dehydrogenase activity were measured to show cytosolic contamination. Enzymatic tests were performed in 96-well plates and measured with a microplate photospectrometer (Epoche 2, BioTek). Fumerase activity measurement was coupled to the reaction of Malate Dehydrogenase (Roche) [67] and NADH production was monitored at 340 nm [67, 68]. The reaction buffer contained 70 mM KH_2_PO4/NaOH pH 7.7, 0.05% Triton X-100, 5 mM MgCl_2_, 2.5 mM NAD, 10 mM sodium fumarate, 10 U/ml Malate Dehydrogenase). NADP: glyceraldehyde 3-phosphate dehydrogenase (GAP-DH) activity was assayed by following the rate of NADPH oxidation at 340 nm [69]. NADP: glucose-6-phosphate dehydrogenase (G6P-DH) was assayed monitoring NADPH production at 340 nm [70]. Alcohol dehydrogenase (ADH) activity was assayed following the production of NADH at 340 nm [71]. Enzyme activity was measured in the following samples: Input (plant extract after filtration, before incubation with beads), supernatant (plant extract after incubation with beads), 3× washing steps (Supernatant of buffer used for washing), beads (washed beads with bound mitochondria, diluted in mitochondria isolation buffer). Additionally, isolated mitochondria bound to beads were quantified using representative microscope images as described for chloroplast quantification.

Protein concentration was determined by Bradford assay [72]. To analyze the amount of protein bound to beads, beads were diluted in water followed by vortexing and sonification (6 10-s bursts) to ensure lysis of all bound organelles.

## Supplementary information

**Additional file 1: Fig. S1.** Transient expression kinetics of organelle-specific SpyTag constructs in *N. benthamiana.* Western Blot analysis of leaf samples transiently expressing HA-tagged organelle-specific SpyTag. Samples were taken every 24 h until 96 h after infiltration (lane 1–5). Lane 1: 0 h, lane 2: 24 h, lane 3: 48 h, lane 4: 72 h and lane 5: 96 h after infiltration. Expression kinetics are shown for (A) Plastid-SpyTag (10 kDa) and (B) Mito-SpyTag (8 kDa). MW: molecular weight (kDa); Black arrows indicate the expected molecular weight.

**Additional file 2: Fig. S2.** Subcellular localization of transiently co-expressed organelle-specific-SpyTag constructs and free eGFP. Organelle-specific-SpyTag constructs and free eGFP were transiently co-expressed in leaves of *Nicotiana benthamiana* plants as negative control to ensure no unspecific interaction between eGFP and SpyTag. CLSM analysis was performed 72 h after infiltration. The expression of free eGFP alone (A–D), in combination with the mitochondria marker IVD-mcherry (I–L) and co-expression with the organelle-specific SpyTag constructs Plastid-SpyTag (E–H), and Mito-SpyTag (M–P) is shown. Autofluorescence of chloroplasts is shown in blue, the mitochondria marker IVD-mcherry is shown in red. Scale bars represent 10 µm.

**Additional file 3: Fig. S3.** Loading efficiency test of SpyCatcher-coated maleimide beads with purified eGFP-SpyTag protein. The loading efficiency test was performed to check bead loading and the functionality of Cys-SpyCatcher immobilized on maleimide beads. Coated and washed beads were incubated with purified, recombinant eGFP-SpyTag protein to allow Catcher/Tag interaction. Incubation of unloaded beads (maleimide beads without SpyCatcher protein) served as negative control. After incubation, supernatant was removed and beads were washed three times. CLSM analysis showed a strong GFP signal on beads that were loaded with SpyCatcher (A), and no signal on beads that lacked SpyCatcher (B), indicating that eGFP-SpyTag can only bind to beads when they are coated with SpyCatcher. Scale bars represent 10 µm.

**Additional file 4: S3.** Sequences.

**Additional file 5: S1.** Cloning scheme.

**Additional file 6: S2.** Primer sequences.

**Additional file 7: Fig. S4.** Time course of heterologous expression of Cysteine-SpyCatcher and eGFP-SpyTag in *E. coli* M15 [pREP4] cells. Expression of recombinant proteins was performed at 28 °C with 180 rpm shaking for 4 h. Expression was induced with IPTG when OD_600_ reached 0.5. Recombinant protein expression of (A) Cys-SpyCatcher (12 kDa) and (B) eGFP-SpyTag ( kDa) was monitored every hour. Samples were adjusted to the same OD_600_ and same volume was loaded on the gel. Samples were boiled in Laemmli buffer prior to SDS-PAGE. Gels were stained with Coomassie Brilliant blue. Lane 1: 0 h (before induction), lane 2-5: 1 h–4 h after induction. Arrows indicate the product bands. MW stands for molecular weight (kDa).

**Additional file 8: Fig. S5.** Purification of recombinantly expressed protein via Ni-NTA affinity chromatography. Ni-NTA affinity chromatography of recombinant 6xHis-tagged proteins under native conditions. Samples were separated by SDS-PAGE. Purification of recombinant (A) Cys-SpyCatcher (12 kDa) and (B) eGFP-SpyTag (30 kDa) is shown. Lane 1: non-induced cells during expression (0 h), lane 2: 4 h induced cells during expression, lane 3: cell pellet after cell lysis (P), lane 4: supernatant after cell lysis (S), lane 5: flow-through after incubation with Ni-NTA (F), lane 6–8: washing steps (in fig. A), lane 6,7: washing steps (in fig. B), lane 9: combined eluate fraction (in fig. A), lane 8–11: fractions 1–4 of the elution step (in fig. B). Same volume of samples was loaded and samples were boiled in Laemmli buffer prior to SDS-PAGE. Gels were stained with Coomassie Brilliant blue. MW stands for molecular weight (kDa). Arrows represent the product band.

**Additional file 9: Fig. S6.** Size exclusion chromatography (SEC) of recombinant Cys-SpyCatcher protein under reducing conditions. Size exclusion chromatography was performed using a Superdex 200 column, Prep Grade 16/60. (A) shows the chromatogram of A280 over elution volume. The arrow indicates the estimated elution position of the void volume. Peak fractions indicated by (*) were analyzed using (B) SDS-PAGE and visualized by Coomassie Brilliant blue staining. Lane 1–9: fractions of SEC, boiled in Laemmli buffer. Same volumes were loaded on the gel. MW stands for molecular weight (kDa).

## Data Availability

All data generated or analysis during this study are included in this published article and its additional information files.

## Abbreviations

ADH: Alcohol dehydrogenase
ATMT: *Agrobacterium tumefaciens* mediated transformation
Plastid-SpyTag: Chloroplast-specific SpyTag (OEP7-SpyTag)
CLSM: Confocal laser scanning microscopy
Co-IP: Co-immunoprecipitation
GS: Glycine-serine (Linker)
IMTACT: Isolation of mitochondria tagged in specific cell types
INTACT: Isolation of nuclei tagged in specific cell types
SEC: Size exclusion chromatography
Mito-SpyTag: Mitochondria-specific SpyTag (NtHxk1-SpyTag)
MW: Molecular weight
MWCO: Molecular weight cutoff
NADP:GAP-DH: NADP-dependent glyceraldehyde-3-phosphate dehydrogenase
NADP:G6P-DH: NADP-dependent glucose-6-phosphate dehydrogenase
