# Supplementary figures and images for: Tagging and catching: rapid isolation and efficient labeling of organelles using the covalent Spy-System in planta
Source: Plant Methods. 2020 Sep 1;16:122. doi: 10.1186/s13007-020-00663-9 (PMC7465787; doi:10.1186/s13007-020-00663-9)

## Slide 1
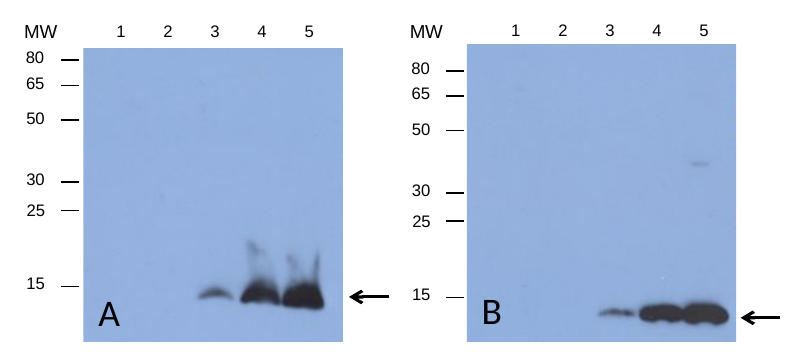

1
2
3
4
5
MW
MW
1
2
3
4
5
80
80
65
65
50
50
30
30
25
25
15
15
B
A

Supplement: Supplementary file 1 — Additional file 1: Fig. S1. Transient expression kinetics of organelle-specific SpyTag constructs in N. benthamiana. Western Blot analysis of leaf samples transiently expressing HA-tagged organelle-specific SpyTag. Samples were taken every 24 h until 96 h after infiltration (lane 1–5). Lane 1: 0 h, lane 2: 24 h, lane 3: 48 h, lane 4: 72 h and lane 5: 96 h after infiltration. Expression kinetics are shown for (A) Plastid-SpyTag (10 kDa) and (B) Mito-SpyTag (8 kDa). MW: molecular weight (kDa); Black arrows indicate the expected molecular weight. [file 13007_2020_663_MOESM1_ESM.pptx]

## Slide 1
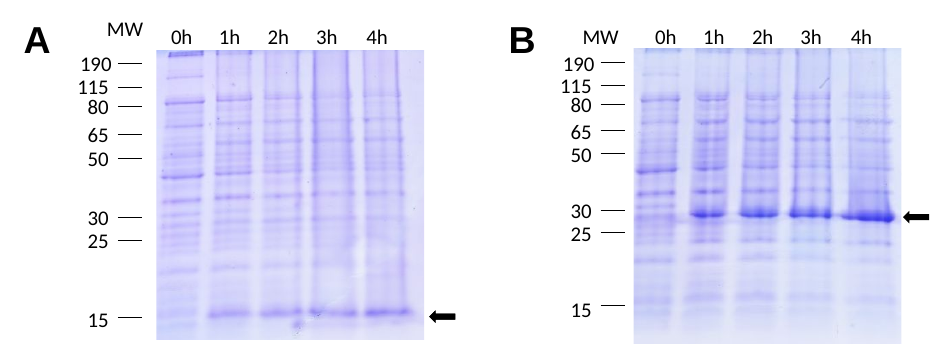

MW
A
B
MW
0h
1h
2h
3h
4h
0h
1h
2h
3h
4h
190
115
80
65
50
30
25
15
190
115
80
65
50
30
25
15

Supplement: Supplementary file 7 — Additional file 7: Fig. S4. Time course of heterologous expression of Cysteine-SpyCatcher and eGFP-SpyTag in E. coli M15 [pREP4] cells. Expression of recombinant proteins was performed at 28 °C with 180 rpm shaking for 4 h. Expression was induced with IPTG when OD600 reached 0.5. Recombinant protein expression of (A) Cys-SpyCatcher (12 kDa) and (B) eGFP-SpyTag ( kDa) was monitored every hour. Samples were adjusted to the same OD600 and same volume was loaded on the gel. Samples were boiled in Laemmli buffer prior to SDS-PAGE. Gels were stained with Coomassie Brilliant blue. Lane 1: 0 h (before induction), lane 2-5: 1 h–4 h after induction. Arrows indicate the product bands. MW stands for molecular weight (kDa). [file 13007_2020_663_MOESM7_ESM.pptx]

## Slide 1
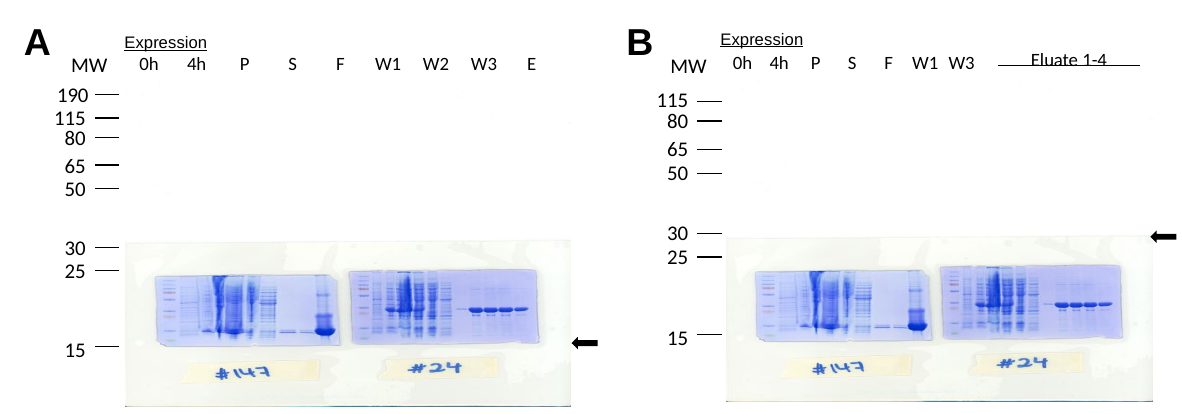

A
B
Expression
Expression
Eluate 1-4
0h
4h
P
S
F
W1
W3
MW
0h
4h
P
S
F
W1
W2
W3
E
MW
190
115
80
65
50
30
25
15
115
80
65
50
30
25
15

Supplement: Supplementary file 8 — Additional file 8: Fig. S5. Purification of recombinantly expressed protein via Ni-NTA affinity chromatography. Ni-NTA affinity chromatography of recombinant 6xHis-tagged proteins under native conditions. Samples were separated by SDS-PAGE. Purification of recombinant (A) Cys-SpyCatcher (12 kDa) and (B) eGFP-SpyTag (30 kDa) is shown. Lane 1: non-induced cells during expression (0 h), lane 2: 4 h induced cells during expression, lane 3: cell pellet after cell lysis (P), lane 4: supernatant after cell lysis (S), lane 5: flow-through after incubation with Ni-NTA (F), lane 6–8: washing steps (in fig. A), lane 6,7: washing steps (in fig. B), lane 9: combined eluate fraction (in fig. A), lane 8–11: fractions 1–4 of the elution step (in fig. B). Same volume of samples was loaded and samples were boiled in Laemmli buffer prior to SDS-PAGE. Gels were stained with Coomassie Brilliant blue. MW stands for molecular weight (kDa). Arrows represent the product band. [file 13007_2020_663_MOESM8_ESM.pptx]

## Slide 1
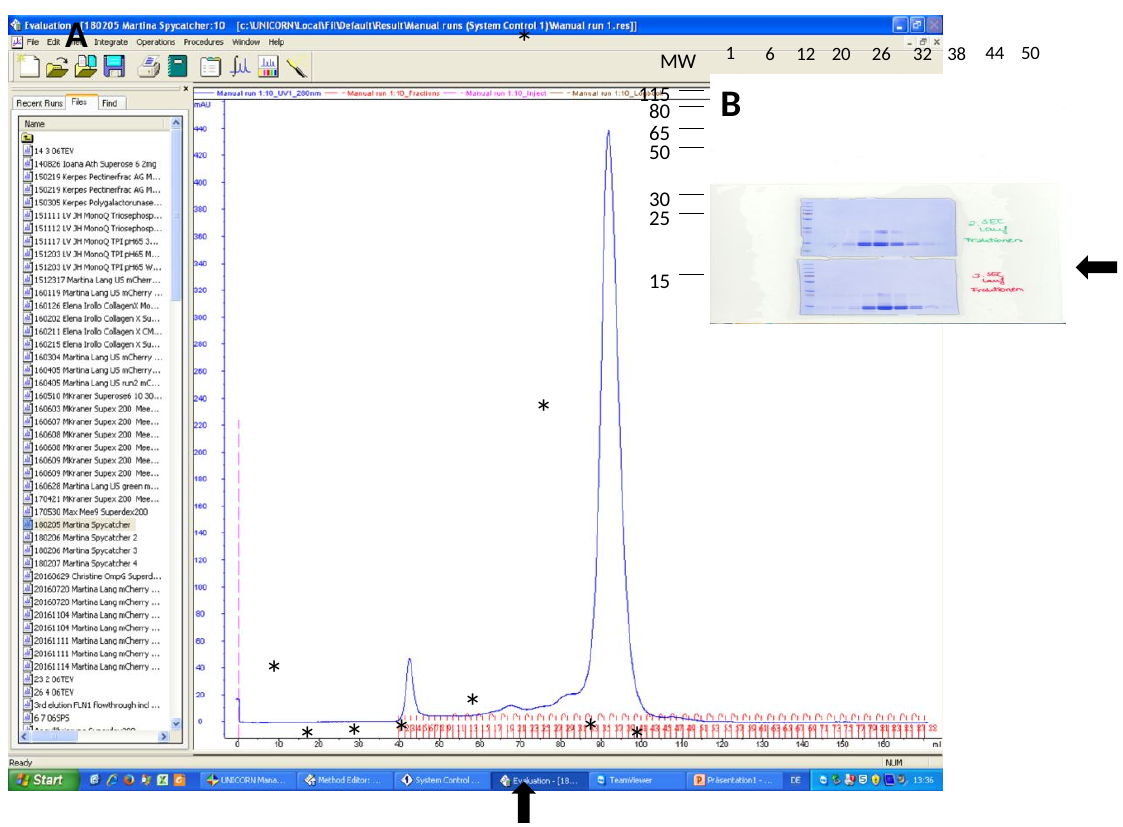

A
*
1
44
50
6
12
20
26
32
38
MW
B
115
80
65
50
30
25
15
*
*
*
*
*
*
*
*

Supplement: Supplementary file 9 — Additional file 9: Fig. S6. Size exclusion chromatography (SEC) of recombinant Cys-SpyCatcher protein under reducing conditions. Size exclusion chromatography was performed using a Superdex 200 column, Prep Grade 16/60. (A) shows the chromatogram of A280 over elution volume. The arrow indicates the estimated elution position of the void volume. Peak fractions indicated by (*) were analyzed using (B) SDS-PAGE and visualized by Coomassie Brilliant blue staining. Lane 1–9: fractions of SEC, boiled in Laemmli buffer. Same volumes were loaded on the gel. MW stands for molecular weight (kDa). [file 13007_2020_663_MOESM9_ESM.pptx]
