# Supplementary material for: Tagging and catching: rapid isolation and efficient labeling of organelles using the covalent Spy-System in planta
Source: Plant Methods. 2020 Sep 1;16:122. doi: 10.1186/s13007-020-00663-9 (PMC7465787; doi:10.1186/s13007-020-00663-9)
Supplement: Supplementary file 2 — Additional file 2: Fig. S2. Subcellular localization of transiently co-expressed organelle-specific-SpyTag constructs and free eGFP. Organelle-specific-SpyTag constructs and free eGFP were transiently co-expressed in leaves of Nicotiana benthamiana plants as negative control to ensure no unspecific interaction between eGFP and SpyTag. CLSM analysis was performed 72 h after infiltration. The expression of free eGFP alone (A–D), in combination with the mitochondria marker IVD-mcherry (I–L) and co-expression with the organelle-specific SpyTag constructs Plastid-SpyTag (E–H), and Mito-SpyTag (M–P) is shown. Autofluorescence of chloroplasts is shown in blue, the mitochondria marker IVD-mcherry is shown in red. Scale bars represent 10 µm. [file 13007_2020_663_MOESM2_ESM.pptx]

## Slide 1
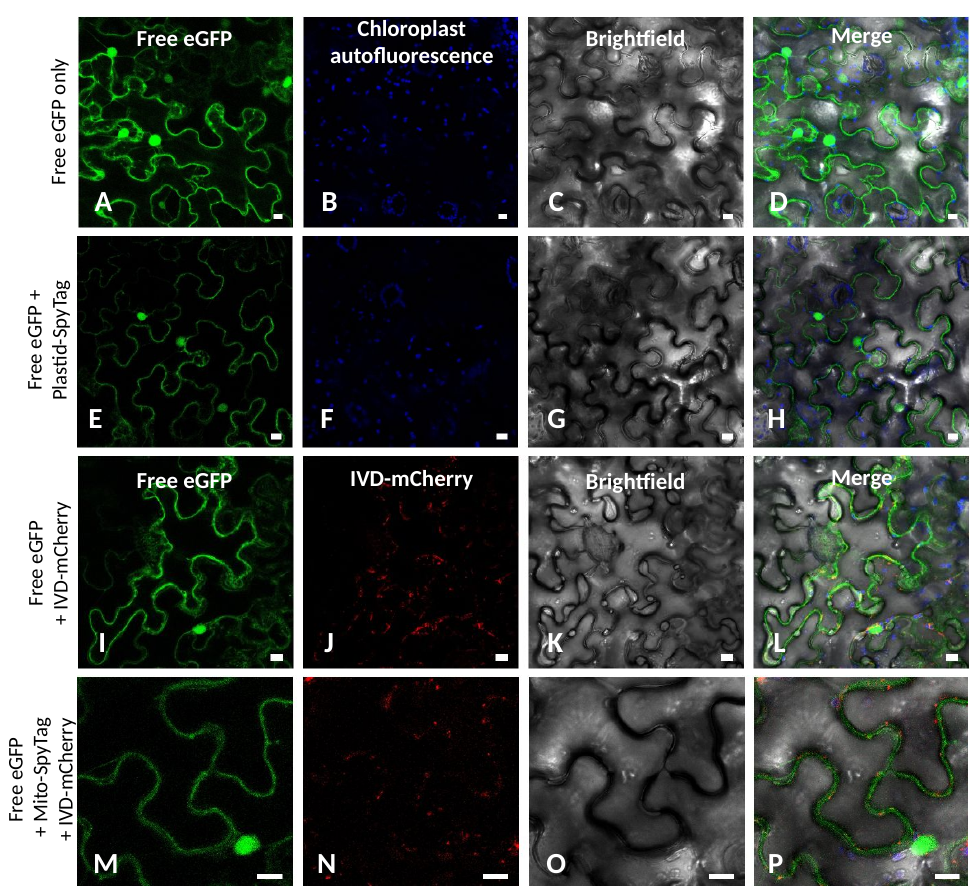

Chloroplast autofluorescence
Merge
Free eGFP
Brightfield
Free eGFP only
A
B
C
D
Free eGFP +
Plastid-SpyTag
E
F
G
H
Merge
IVD-mCherry
Free eGFP
Brightfield
Free eGFP
+ IVD-mCherry
I
J
K
L
Free eGFP
 + Mito-SpyTag
+ IVD-mCherry
M
N
O
P
