# Supplementary material for: Tagging and catching: rapid isolation and efficient labeling of organelles using the covalent Spy-System in planta
Source: Plant Methods. 2020 Sep 1;16:122. doi: 10.1186/s13007-020-00663-9 (PMC7465787; doi:10.1186/s13007-020-00663-9)
Supplement: Supplementary file 3 — Additional file 3: Fig. S3. Loading efficiency test of SpyCatcher-coated maleimide beads with purified eGFP-SpyTag protein. The loading efficiency test was performed to check bead loading and the functionality of Cys-SpyCatcher immobilized on maleimide beads. Coated and washed beads were incubated with purified, recombinant eGFP-SpyTag protein to allow Catcher/Tag interaction. Incubation of unloaded beads (maleimide beads without SpyCatcher protein) served as negative control. After incubation, supernatant was removed and beads were washed three times. CLSM analysis showed a strong GFP signal on beads that were loaded with SpyCatcher (A), and no signal on beads that lacked SpyCatcher (B), indicating that eGFP-SpyTag can only bind to beads when they are coated with SpyCatcher. Scale bars represent 10 µm. [file 13007_2020_663_MOESM3_ESM.pptx]

## Slide 1
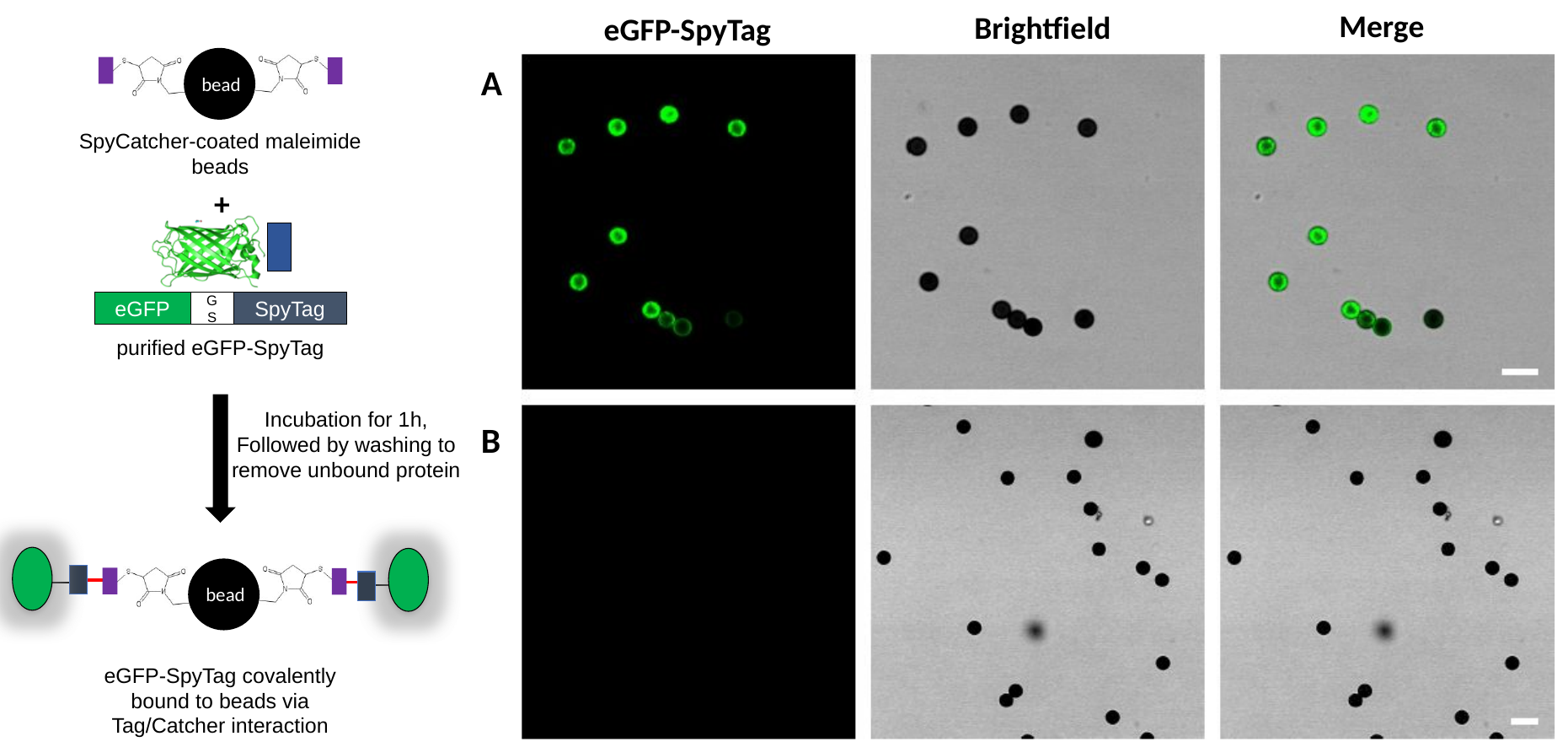

Merge
Brightfield
eGFP-SpyTag
A
B
bead
SpyCatcher-coated maleimide beads
+
eGFP
GS
SpyTag
purified eGFP-SpyTag
Incubation for 1h,
Followed by washing to remove unbound protein
bead
eGFP-SpyTag covalently bound to beads via Tag/Catcher interaction
