# Supplementary material for: Tagging and catching: rapid isolation and efficient labeling of organelles using the covalent Spy-System in planta
Source: Plant Methods. 2020 Sep 1;16:122. doi: 10.1186/s13007-020-00663-9 (PMC7465787; doi:10.1186/s13007-020-00663-9)
Supplement: Supplementary file 4 — Additional file 4: S3. Sequences. [file 13007_2020_663_MOESM4_ESM.docx]

**pRB-35S expression constructs (plant)**

**Plastid-SpyTag (Kozak-OEP7-GS-SpyTag-HA):**

GGATCCAACAATGGGAAAAACTTCTGGAGCTAAGCAAGCAACTGTTGTGGTAGCAGCTATGGCTTTAGGATGGTTAGCAATTGAGATCGCTTTCAAGCCTTTTCTTGATAAGTTCCGTTCCTCAATTGACAAGTCTGACCCAACTAAAGACCCAGATGACTTCGACACTGCAGCTACTGCAACTACATCCAAGGAGGGATTGGGATCTGGTTCAGGCTCCGGAGCGCATATTGTGATGGTGGATGCGTATAAACCGACCAAATACCCTTATGATGTGCCAGACTACGCATAAGTCGAC

MGKTSGAKQATVVVAAMALGWLAIEIAFKPFLDKFRSSIDKSDPTKDPDDFDTAATATTSKEGLGSGSGSGAHIVMVDAYKPTKYPYDVPDYA*

**Mito-SpyTag (Omega-NtHxk1-GS-SpyTag-HA)**

GGATCCGGTACCTTTACAACAATTACCAACAACAACAAACAACAAACAACATTACAATTACTATTTACAATTACCATGAAGAAAGCGACGGTGGGAGCCGCCGTAATTGGCGCCGCTACGGTATGTGCAGTGGCGGCATTAATAGTGAACCACCGTATGCGCAAATCTAGCAAATGGGCACGTGCTATGGCTATTCTTCGTGGATCTGGTTCAGGCTCCGGAGCGCATATTGTGATGGTGGATGCGTATAAACCGACCAAATACCCTTATGATGTGCCAGACTACGCATAAGTCGAC

MKKATVGAAVIGAATVCAVAALIVNHRMRKSSKWARAMAILRGSGSGSGAHIVMVDAYKPTKYPYDVPDYA*

**Kozak-eGFP-GS-SpyCatcher-HA**

GGATCCAACAATGGTGAGCAAGGGCGAGGAGCTGTTCACCGGGGTGGTGCCCATCCTGGTCGAGCTGGACGGCGACGTAAACGGCCACAAGTTCAGCGTGTCCGGCGAGGGCGAGGGCGATGCCACCTACGGCAAGCTGACCCTGAAGTTCATCTGCACCACCGGCAAGCTGCCCGTGCCCTGGCCCACCCTCGTGACCACCCTGACCTACGGCGTGCAGTGCTTCAGCCGCTACCCCGACCACATGAAGCAGCACGACTTCTTCAAGTCCGCCATGCCCGAAGGCTACGTCCAGGAGCGCACCATCTTCTTCAAGGACGACGGCAACTACAAGACCCGCGCCGAGGTGAAGTTCGAGGGCGACACCCTGGTGAACCGCATCGAGCTGAAGGGCATCGACTTCAAGGAGGACGGCAACATCCTGGGGCACAAGCTGGAGTACAACTACAACAGCCACAACGTCTATATCATGGCCGACAAGCAGAAGAACGGCATCAAGGTGAACTTCAAGATCCGCCACAACATCGAGGACGGCAGCGTGCAGCTCGCCGACCACTACCAGCAGAACACCCCCATCGGCGACGGCCCCGTGCTGCTGCCCGACAACCACTACCTGAGCACCCAGTCCGCCCTGAGCAAAGACCCCAACGAGAAGCGCGATCACATGGTCCTGCTGGAGTTCGTGACCGCCGCCGGGATCACTCTCGGCATGGACGAGCTGTACAAGGGATCTGGTTCAGGCTCCGGAGAAGAAGATAGTGCTACCCATATTAAATTCTCAAAACGTGATGAGGACGGCAAAGAGTTAGCTGGTGCAACTATGGAGTTGCGTGATTCATCTGGTAAAACTATTAGTACATGGATTTCAGATGGACAAGTGAAAGATTTCTACCTGTATCCAGGAAAATATACATTTGTCGAAACCGCAGCACCAGACGGTTATGAGGTAGCAACTGCTATTACCTTTACAGTTAATGAGCAAGGTCAGGTTACTGTAAATGGCAAAGCAACTAAAGGTTACCCTTATGATGTGCCAGACTACGCATAAGTCGAC

MVSKGEELFTGVVPILVELDGDVNGHKFSVSGEGEGDATYGKLTLKFICTTGKLPVPWPTLVTTLTYGVQCFSRYPDHMKQHDFFKSAMPEGYVQERTIFFKDDGNYKTRAEVKFEGDTLVNRIELKGIDFKEDGNILGHKLEYNYNSHNVYIMADKQKNGIKVNFKIRHNIEDGSVQLADHYQQNTPIGDGPVLLPDNHYLSTQSALSKDPNEKRDHMVLLEFVTAAGITLGMDELYKGSGSGSGEEDSATHIKFSKRDEDGKELAGATMELRDSSGKTISTWISDGQVKDFYLYPGKYTFVETAAPDGYEVATAITFTVNEQGQVTVNGKATKGYPYDVPDYA*

**pQE-9 expression constructs (bacteria)**

**Cys-SpyCatcher (Cystein-TEV-SpyCatcher)**

CCTGGGATCCTGCGGCAGCGAAAACCTGTATTTTCAGGGTTCTGAAGAAGATAGTGCTACCCATATTAAATTCTCAAAACGTGATGAGGACGGCAAAGAGTTAGCTGGTGCAACTATGGAGTTGCGTGATTCATCTGGTAAAACTATTAGTACATGGATTTCAGATGGACAAGTGAAAGATTTCTACCTGTATCCAGGAAAATATACATTTGTCGAAACCGCAGCACCAGACGGTTATGAGGTAGCAACTGCTATTACCTTTACAGTTAATGAGCAAGGTCAGGTTACTGTAAATGGCAAAGCAACTAAAGGTTAAGTCGACCCTG

HHHHHHCGSENLYFQGSEEDSATHIKFSKRDEDGKELAGATMELRDSSGKTISTWISDGQVKDFYLYPGKYTFVETAAPDGYEVATAITFTVNEQGQVTVNGKATKG*

**eGFP-GS-SpyTag**

CCTGGGATCCGTGAGCAAGGGCGAGGAGCTGTTCACCGGGGTGGTGCCCATCCTGGTCGAGCTGGACGGCGACGTAAACGGCCACAAGTTCAGCGTGTCCGGCGAGGGCGAGGGCGATGCCACCTACGGCAAGCTGACCCTGAAGTTCATCTGCACCACCGGCAAGCTGCCCGTGCCCTGGCCCACCCTCGTGACCACCCTGACCTACGGCGTGCAGTGCTTCAGCCGCTACCCCGACCACATGAAGCAGCACGACTTCTTCAAGTCCGCCATGCCCGAAGGCTACGTCCAGGAGCGCACCATCTTCTTCAAGGACGACGGCAACTACAAGACCCGCGCCGAGGTGAAGTTCGAGGGCGACACCCTGGTGAACCGCATCGAGCTGAAGGGCATCGACTTCAAGGAGGACGGCAACATCCTGGGGCACAAGCTGGAGTACAACTACAACAGCCACAACGTCTATATCATGGCCGACAAGCAGAAGAACGGCATCAAGGTGAACTTCAAGATCCGCCACAACATCGAGGACGGCAGCGTGCAGCTCGCCGACCACTACCAGCAGAACACCCCCATCGGCGACGGCCCCGTGCTGCTGCCCGACAACCACTACCTGAGCACCCAGTCCGCCCTGAGCAAAGACCCCAACGAGAAGCGCGATCACATGGTCCTGCTGGAGTTCGTGACCGCCGCCGGGATCACTCTCGGCATGGACGAGCTGTACAAGGGATCTGGTTCAGGCTCCGGAGCGCATATTGTGATGGTGGATGCGTATAAACCGACCAAATAACTGCAGCCTG

HHHHHHGSVSKGEELFTGVVPILVELDGDVNGHKFSVSGEGEGDATYGKLTLKFICTTGKLPVPWPTLVTTLTYGVQCFSRYPDHMKQHDFFKSAMPEGYVQERTIFFKDDGNYKTRAEVKFEGDTLVNRIELKGIDFKEDGNILGHKLEYNYNSHNVYIMADKQKNGIKVNFKIRHNIEDGSVQLADHYQQNTPIGDGPVLLPDNHYLSTQSALSKDPNEKRDHMVLLEFVTAAGITLGMDELYKGSGSGSGAHIVMVDAYKPTK*
