# Supplementary material for: Tagging and catching: rapid isolation and efficient labeling of organelles using the covalent Spy-System in planta
Source: Plant Methods. 2020 Sep 1;16:122. doi: 10.1186/s13007-020-00663-9 (PMC7465787; doi:10.1186/s13007-020-00663-9)
Supplement: Supplementary file 5 — Additional file 5: S1. Cloning scheme. [file 13007_2020_663_MOESM5_ESM.docx]

**Cloning scheme**

**Plastid-SpyTag (BamHI-Kozak-OEP7-GS-SpyTag-HA-TAA-SalI)**

**FP_OEP7:** 5´ - GGATCCAACAATGGGAAAAACTTCTGGAGCTAAGC – 3`

**RP_OEP 7**: 5´- GTCGACTTATGCGTAGTCTGGCACATCATAAGGGTATTTGGTCG

GTTTATACGCATCCACCATCACAATATGCGCTCCGGAGCCTGAACCAGATCC -3´

Template: *OEP7* from At2g52420

**eGFP-SpyCatcher (BamHI-Kozak-eGFP-GS-SpyCatcher-HA-TAA-SalI)**

PCR1:

**FP_eGFP:** 5´ - GGATCCAACAATGGTGAGCAAGGGCGAGGAGCTG– 3´

**RP_eGFP:** 5´ - CTTCTCCGGAGCCTGAACCAGATCCCTTGTACAGCTCGTCCAT GCCGAGAG – 3`

Template: eGFP (Karimi et al., 2005)

PCR2:

**FP_SC:** 5` - CTGTACAAGGGATCTGGTTCAGGCTCCGGAGAAGAAGATAGTGCTACCC - 3`

**RP_SC:** 5’- GTCGACTTATGCGTAGTCTGGCACATCATAAGGGTAACCTTTAGTTG

CTTTGCCATTTAC - 3’

Template: SpyCatcher

PCR3-overlap:

**FP_eGFP + RP_SC**

Templates: PCR products (PCR1 + PCR2)

**Cys-SpyCatcher (BamHI-Cystein-TEV-SpyCatcher-TAA-SalI)**

**FP_CSC:** 5’-CCTGGGATCCTGCGGCAGCGAAAACCTGTATTTTCAGGGTTCTGAA

GAAGATAGTGCTACCCATATTAAATTC 3´

**RP_CSC:** 5´ - CAGGGTCGACTTAACCTTTAGTTGCTTTGCCATTTACAG - 3´

Template: SpyCatcher

**eGFP-SpyTag (BamHI-eGFP-GS-SpyTag-TAA-PstI)**

**FP_eGFP-ST:** 5´ - CCTGGGATCCGTGAGCAAGGGCGAGGAGCTGTTC - 3´

**RP_eGFP-ST:** 5´ - CAGGCTGCAGTTATTTGGTCGGTTTATACGCATCCACCATC

ACAATATGCGCTCCGGAGCCTGAACCAGATCC - 3´

Template: eGFP-SpyCatcher
