# Supplementary material for: Tagging and catching: rapid isolation and efficient labeling of organelles using the covalent Spy-System in planta
Source: Plant Methods. 2020 Sep 1;16:122. doi: 10.1186/s13007-020-00663-9 (PMC7465787; doi:10.1186/s13007-020-00663-9)
Supplement: Supplementary file 6 — Additional file 6: S2. Primer sequences. [file 13007_2020_663_MOESM6_ESM.docx]

| **FP_OEP7** | 5´ - GGATCCAACAATGGGAAAAACTTCTGGAGCTAAGC – 3` |
| --- | --- |
| **RP_OEP** | 5´-GTCGACTTATGCGTAGTCTGGCACATCATAAGGGTATTT  GGTCGGTTTATACGCATCCACCATCACAATATGCGCTCCGGA  GCCTGAACCAGATCC - 3´ |
| **FP_eGFP** | 5´ - GGATCCAACAATGGTGAGCAAGGGCGAGGAGCTG– 3´ |
| **RP_eGFP** | 5´ - CTTCTCCGGAGCCTGAACCAGATCCCTTGTACAGCTCGT  CCATGCCGAGAG – 3` |
| **FP_SC** | 5` - CTGTACAAGGGATCTGGTTCAGGCTCCGGAGAAGAAGA  TAGTGCTACCC – 3` |
| **RP_SC** | 5’-GTCGACTTATGCGTAGTCTGGCACATCATAAGGGTAACCT  TTAGTTGCTTTGCCATTTAC -3’ |
| **FP_CSC** | 5’-CCTGGGATCCTGCGGCAGCGAAAACCTGTATTTTCAGGG  TTCTGAAGAAGATAGTGCTACCCATATTAAATTC 3´ |
| **RP_CSC** | 5´ - CAGGGTCGACTTAACCTTTAGTTGCTTTGCCAT  TTACAG - 3´ |
| **FP_eGFP-ST** | 5´ - CCTGGGATCCGTGAGCAAGGGCGAGGAGCTGTTC - 3´ |
| **RP_eGFP-ST** | 5´ - CAGGCTGCAGTTATTTGGTCGGTTTATACGCATCC  ACCATCACAATATGCGCTCCGGAGCCTGAACCAGATCC - 3´ |

**Primer**
